# Refractory testicular germ cell tumors are highly sensitive to the second generation DNA methylation inhibitor guadecitabine

**DOI:** 10.18632/oncotarget.13811

**Published:** 2016-12-07

**Authors:** Costantine Albany, Mary P. Hever-Jardine, Katherine M. von Herrmann, Christina Y. Yim, Janice Tam, Joshua M. Warzecha, Leah Shin, Sarah E. Bock, Brian S. Curran, Aneeq S. Chaudhry, Fred Kim, George E. Sandusky, Pietro Taverna, Sarah J. Freemantle, Brock C. Christensen, Lawrence H. Einhorn, Michael J. Spinella

**Affiliations:** ^1^ Division of Hematology/Oncology, Melvin and Bren Simon Cancer Center, Indiana University School of Medicine, Indianapolis, IN, USA; ^2^ Departments of Pharmacology and Toxicology and Epidemiology, Geisel School of Medicine at Dartmouth, Hanover, NH, USA; ^3^ Astex Pharmaceutical, Pleasanton, CA, USA; ^4^ Department of Comparative Biosciences, The University of Illinois at Urbana-Champaign, Urbana, IL, USA; ^5^ Department of Pathology, Melvin and Bren Simon Cancer Center, Indiana University School of Medicine, Indianapolis, IN, USA; ^6^ Department of Epidemiology, Geisel School of Medicine at Dartmouth, Hanover, NH, USA

**Keywords:** testicular cancer, embryonal carcinoma, DNA methylation, SGI-110, in vivo

## Abstract

Testicular germ cell tumors (TGCTs) are the most common cancers of young males. A substantial portion of TGCT patients are refractory to cisplatin. There are no effective therapies for these patients, many of whom die from progressive disease. Embryonal carcinoma (EC) are the stem cells of TGCTs. In prior *in vitro* studies we found that EC cells were highly sensitive to the DNA methyltransferase inhibitor, 5-aza deoxycytidine (5-aza). Here, as an initial step in bringing demethylation therapy to the clinic for TGCT patients, we evaluated the effects of the clinically optimized, second generation demethylating agent guadecitabine (SGI-110) on EC cells in an animal model of cisplatin refractory testicular cancer. EC cells were exquisitely sensitive to guadecitabine and the hypersensitivity was dependent on high levels of DNA methyltransferase 3B. Guadecitabine mediated transcriptional reprogramming of EC cells included induction of p53 targets and repression of pluripotency genes. As a single agent, guadecitabine completely abolished progression and induced complete regression of cisplatin resistant EC xenografts even at doses well below those required to impact somatic solid tumors. Low dose guadecitabine also sensitized refractory EC cells to cisplatin *in vivo*. Genome-wide analysis indicated that *in vivo* antitumor activity was associated with activation of p53 and immune-related pathways and the antitumor effects of guadecitabine were dependent on p53, a gene rarely mutated in TGCTs. These preclinical findings suggest that guadecitabine alone or in combination with cisplatin is a promising strategy to treat refractory TGCT patients.

## INTRODUCTION

Testicular germ cell tumors (TGCTs) are the most common cancer in men 15 to 35 with increasing incidence in the last 30 years [1]. Testicular cancer patients are successfully treated with a combination of cisplatin, bleomycin and etoposide [2, 3]. However, 15–20% of all patients and 50% of patients with poor-risk disease are refractory to treatment and many eventually die from progressive disease [4–6]. Further, there are patients who initially respond to therapy but undergo late relapse. These patients are rarely cured if their disease is not amenable to surgical resection [7, 8]. Therapies to treat the cisplatin resistant population is a major un-met clinical need. Unlike most other cancers, morbidity and mortality due to testicular cancer occurs during the most productive years of a patient's life. An average of more than 35 years of life is lost when a testicular cancer death occurs, well over a decade longer than any other adult malignancy. Further, there is a need to reduce co-morbidities and the burden of therapy-related toxicities and survivorship issues [9–10].

TGCTs consist of two histologically distinct subtypes: seminomas (40%) and nonseminomas (60%). Nonseminomas are further divided into embryonal carcinoma (EC), teratoma, yolk sac tumor and choriocarcinoma [11]. TGCTs are thought to arise from transformation of primordial germ cells and pluripotent EC represents the stem cell component of nonseminoma and can differentiate into mature nonseminoma subtypes [12]. One of the unique features of TGCTs is distinct genome-wide DNA methylation compared to somatic solid tumors that is proposed to be linked to their pluripotent nature and primordial germ cell origins. Most human cancers have global DNA hypomethylation coupled with hypermethylation of CpG islands at specific tumor suppressor gene promoters [13]. Seminomas appear to have greatly reduced levels of overall DNA methylation as compared to adult somatic tumors while nonseminomas and EC possess intermediate levels of DNA methylation [14, 15].

DNA methylation is maintained primarily by the DNA methyltransferase, DNMT1, while *de novo* DNA methylation is mediated primarily by DNMT3A and DNMT3B [16]. The nucleoside analogs 5-aza-deoxcytidine (referred to here as 5-aza) and 5-aza-cytidine are potent DNA methyltransferase inhibitors (DNMTIs) [17]. We showed that EC cells are highly sensitive to low concentrations of 5-aza *in vitro* [18]. This sensitivity appeared to partially dependent on high expression of the pluripotency-associated methyltransferase, DNMT3B [18].

In the current study, we evaluate the effects of the clinically and pharmacological optimized demethylating agent guadecitabine (SGI-110) on EC cells and in an animal model of cisplatin refractory nonseminoma testicular cancer [19]. EC-derived cisplatin resistant cells and tumors were highly sensitive to guadecitabine and *in vivo* guadecitabine was also able to sensitize cisplatin resistant tumors to cisplatin. Further, we demonstrate that these antitumor effects are highly associated with activation of p53, a gene rarely mutated in TGCTs. Interestingly, immune pathway genes were also induced in EC tumors by guadecitabine, suggesting that tumor immune activation could enhance antitumor activity in the clinic. Together our findings provide strong rationale for further development of guadecitabine as a novel therapy to treat patients with cisplatin-refractory testicular cancer.

## RESULTS

### Cisplatin sensitive and resistant EC cells are highly sensitive to low concentrations of guadecitabine in a DNMT3B-dependent manner

**Figure 1:**
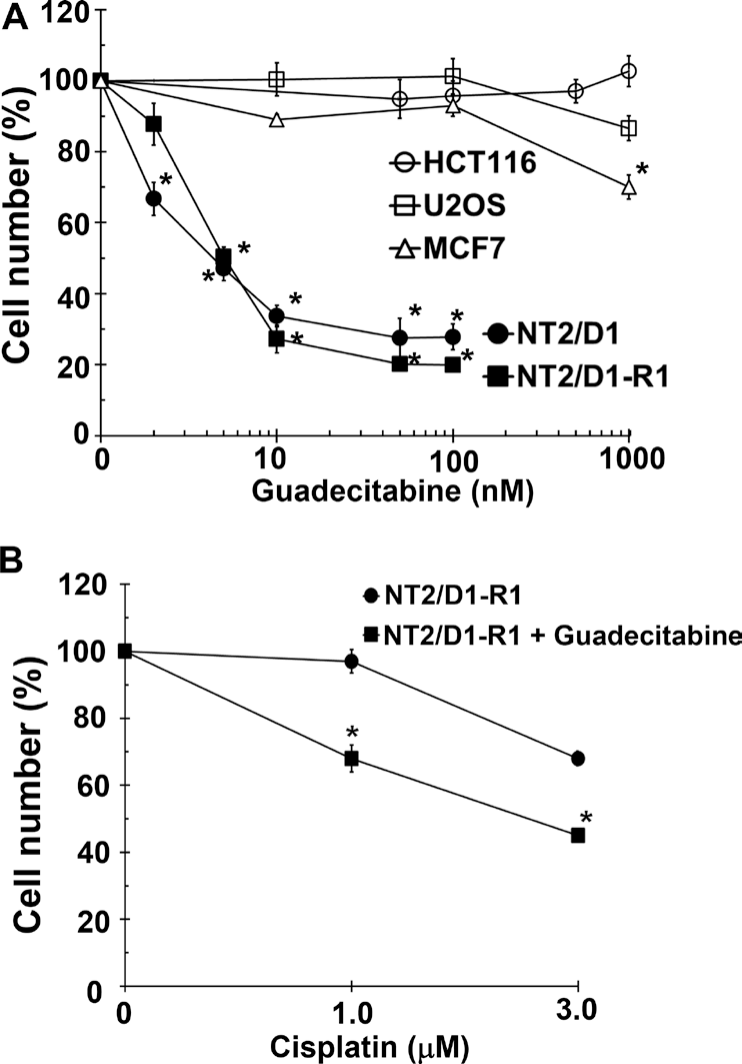
EC cells are highly sensitive to low concentrations of guadecitabine (**A**) Cisplatin sensitive EC cells, NT2/D1, and cisplatin resistant cells, NT2/D1-R1, but not HCT116 colon cancer cells, U2OS osteosarcoma cells, or MCF7 breast cancer cells are sensitive to low concentrations of guadecitabine. Guadecitabine was added for 3 days to exponentially growing cultures. Viable cell growth and survival were measured. All data points are the average of biological triplicates. Error bars are standard deviation. *p < 0.01 comparing drug treatments to vehicle control in the same cell line. (**B**) Pretreatment with low concentrations of guadecitabine restores cisplatin sensitivity to cisplatin resistant EC cells. NT2/D1-R1 cells were pretreated with vehicle or 10 nM guadecitabine for 3 days before replating and a 48-hour recovery period followed by indicated cisplatin treatments for 6 hours. Cell viability was measured 3 days later. All data points are the average of biological triplicates. Error bars are standard deviation. *p < 0.01 comparing NTD1-R1 to NT2D1-R1 + guadecitabine. Experiments were repeated twice with similar results.

**Figure 2:**
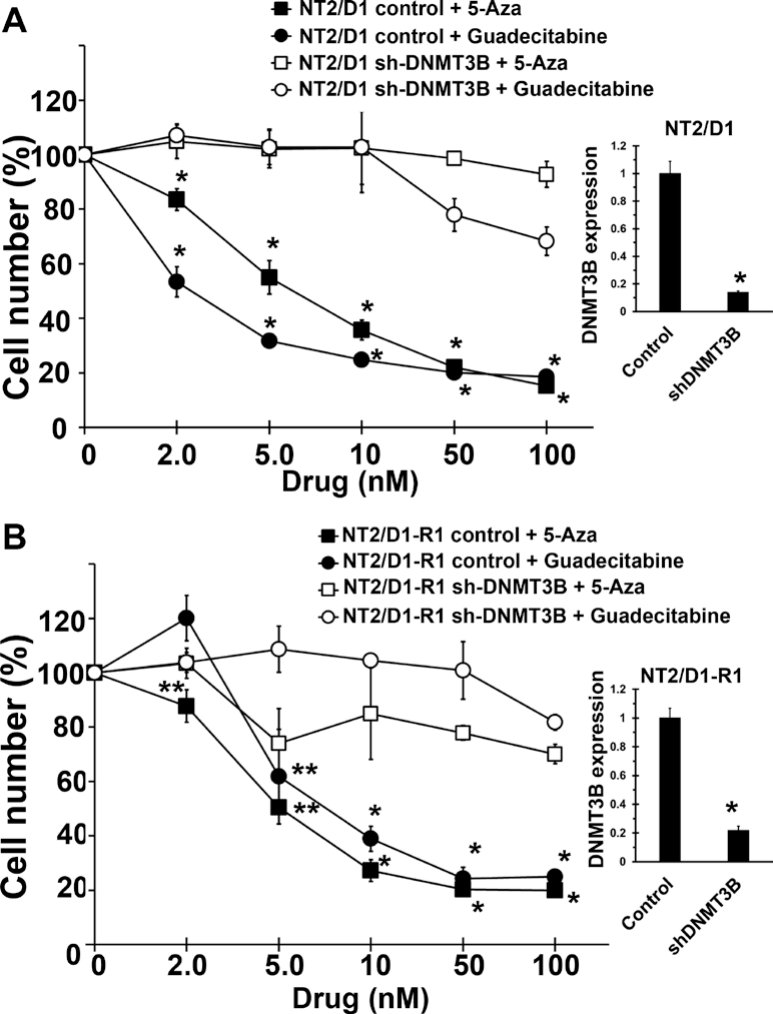
Guadecitabine and 5-aza sensitivity in EC cells are dependent on DNMT3B DNMT3B knockdown results in resistance to guadecitabine and 5-aza in (**A**) NT2/D1 and (**B**) NT2/D1-R1 cells. Guadecitabine or 5-aza was added for 3 days to exponentially growing cultures. Viable cell growth and survival were measured. All data points are the average of biological triplicates. Error bars are standard deviation. *p < 0.01 and **p < 0.05 comparing control cell lines with their corresponding shDNMT3B cells. Right, The shDNMT3B cells were previously characterized and have a knockdown at the protein level of greater than 90% (18) and knockdown was further confirmed by real-time PCR assays. Error bars are standard deviation. *p < 0.01 comparing control cell lines with their corresponding shDNMT3B cells. Experiments were repeated twice with similar results.

We previously demonstrated that a variety of TGCT-derived EC cells lines are highly sensitive to low nanomolar concentrations of 5-aza [18]. However, 5-aza and other DNA methylation inhibitors (DNMTIs) are subject to rapid degradation by hydrolytic cleavage and deamination necessitating chronic intravenous infusion [20]. In anticipation of potential clinical assessment of demethylation therapy for TGCTs we assessed the effects of the second generation demethylating agent guadecitabine that is not subject to the same metabolism as other DNMTIs and can be given subcutaneously with a longer effective half-life and a more extended exposure window compared to 5-aza [19]. Cisplatin sensitive EC cells, NT2/D1 and cisplatin resistant NT2/D1-R1 cells were highly sensitive to guadecitabine with an IC-50 of 5 nM (Figure 1A). This is in contrast to the effects of guadecitabine on somatic solid tumor cells HCT116, U20S and MCF7 that were relatively insensitive to guadecitabine at concentrations as high at 1 μM. Further, pretreatment of cisplatin resistant NT2/D1-R1 cells with low concentrations of guadecitabine resensitized the cells to cisplatin (Figure 1B). In this experiment cells pretreated with guadecitabine were allowed to recover before treating with cisplatin such that the cells had a comparable growth rate to cells not pretreated with guadecitabine. We have linked 5-aza hypersensitivity in EC cells to high levels of the DNA methyltransferase, DNMT3B and provided evidence to suggest that the relative insensitivity of somatic cancer cells to 5-aza is due to low DNA methyltransferase levels and activity [18]. The sensitivity of cisplatin sensitive and resistant EC cells to guadecitabine was highly dependent on DNMT3B as DNMT3B knockdown results in robust guadecitabine resistance in NT2/D1 and NT2/D1-R1 cells (Figure 2). These data suggest that EC cells are exquisitely sensitive to the novel DNMTI guadecitabine, in part due to high levels of DNMT3B.

### Low concentrations of guadecitabine transcriptionally reprograms cisplatin sensitive and resistant EC cells

**Figure 3:**
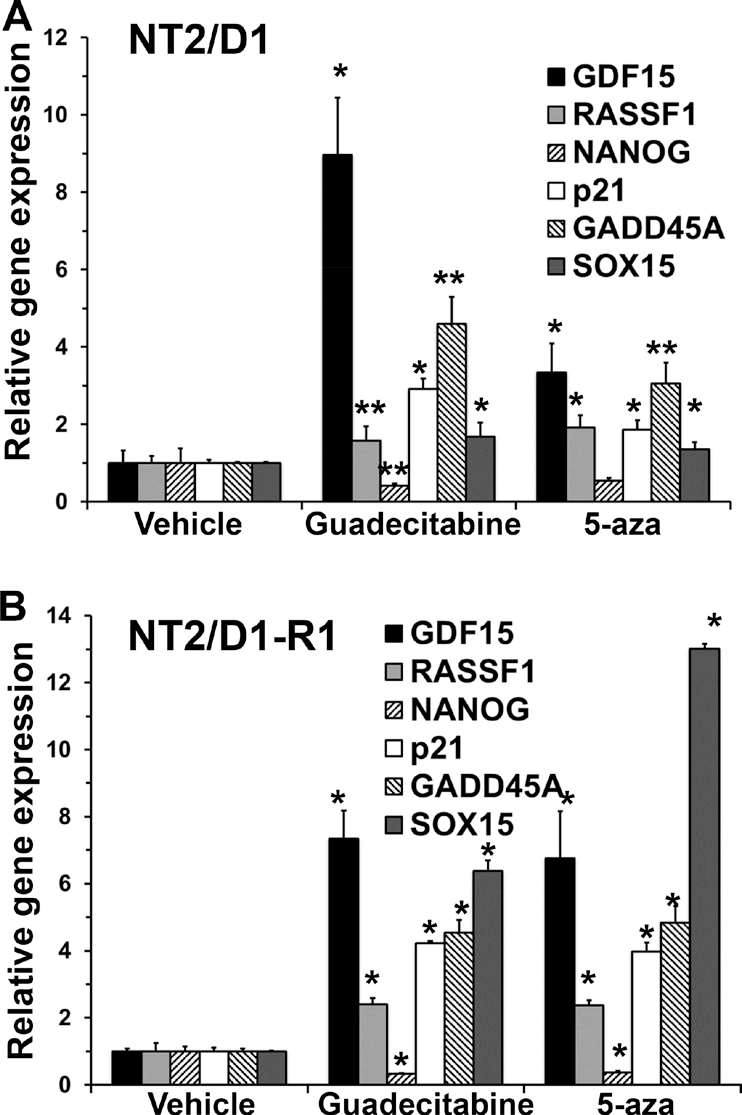
Guadecitabine and 5-aza remodel gene transcription in EC cells (**A**) NT2/D1 and (**B**) NT2/D1-R1 cells were treated with 10 nM 5-aza or guadecitabine for 3 days and RNA was harvested for real-time PCR analysis. Each bar represents the average of 3 biological replicates. Error bars are standard deviation. *p < 0.01 and **p < 0.05 comparing each drug treatment to vehicle treatment. Experiments were repeated twice with similar results.

We assessed expression of genes determined to be target genes of 5-aza in EC cells. P53 target genes GDF15, p21 and GADD45A were induced in response to guadecitabine while the pluripotency gene NANOG was repressed in both NT2/D1 and NT2/D1-R1 cells (Figure 3). In addition, a gene known to be highly methylated in TGCTs, RASSF1, and a gene we identified as a novel methylated gene in EC cells, SOX15 [21], were both induced with 5-aza and guadecitabine (Figure 3). Taken together these data strongly suggest guadecitabine potently suppresses the *in vitro* proliferation and survival of EC cells at low nanomolar concentrations in a manner similar to 5-aza [18, 21]. It is of note that our data does not prove that the identified gene expression changes are responsible for the anti-proliferation and anti-survival activity of guadecitabine, which will require detailed follow-up studies.

### Guadecitabine displays potent antitumor effects *in vivo* toward cisplatin resistant EC

**Figure 4:**
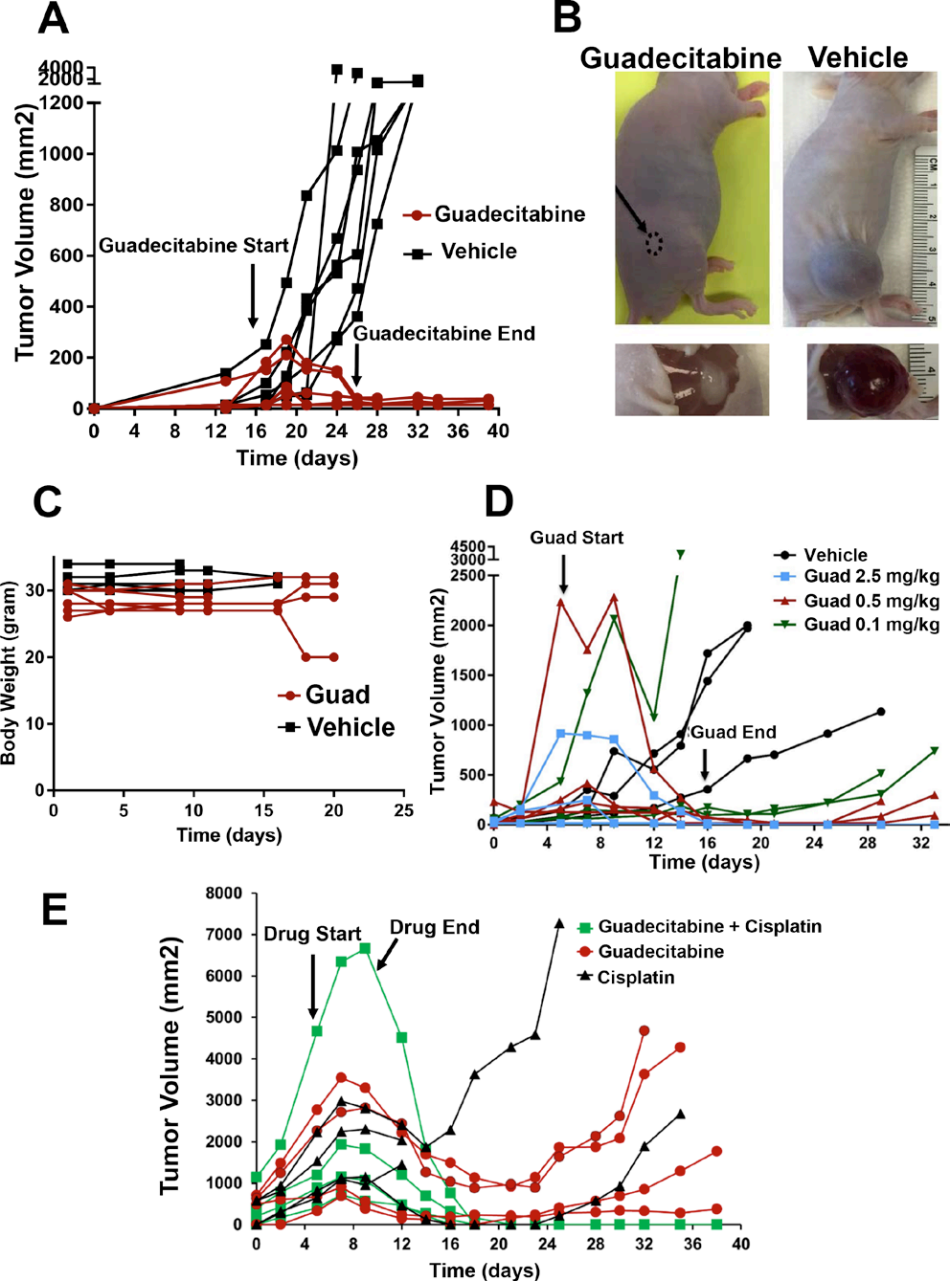
Guadecitabine potently inhibits growth and induces regression of cisplatin resistant EC tumors *in vivo* (**A**) Mice bearing cisplatin resistant tumors were randomized to subcutaneous injections of guadecitabine at a dose of 2.0 mg/kg or vehicle control. Randomization and injections were performed when palpable tumors were just evident. Treatments were for 5 days per week for 2 weeks. Each line represents a single mouse (7 vehicle treated, 7 guadecitabine treated). (**B**) Representative tumors from the experiment in A. In most cases, tumors completely regressed after guadecitabine therapy while in a few cases a residual remnant tumor remained. (**C**) Treatment of 2.0 mg/kg guadecitabine for 5 days per week for 2 weeks did not effect whole body weight. Measurements began on the day after the first guadecitabine injection. (**D**) Mice bearing cisplatin resistant tumors were randomized to vehicle control or indicated dosages of guadecitabine for 5 days per week for 2 weeks. Each line represents a single mouse (3 mice per group). Note: some tumors were larger than just palpable size at the start of therapy and still regressed completely with guadecitabine; however, one mouse at the lowest, 0.1 mg/kg dose had only a partial response. (**E**) Low dose guadecitabine and cisplatin combination therapy is effective for cisplatin resistant EC cells. Mice bearing cisplatin resistant tumors were randomized to 3 groups. Guadecitabine alone at a dose of 0.5 mg/kg for 5 days for only 1 week, guadecitabine at a dose of 0.5 mg/kg for 5 days followed two days later with a single dose of 9 mg/kg cisplatin, or two doses of 9 mg/kg cisplatin on consecutive days. Each line represents a single mouse (4 mice per group). Note that two-dose cisplatin treatment alone resulted in lethal toxicity in two mice on day 12. No overt toxicity was seen in guadecitabine or guadecitabine + single dose cisplatin groups.

In myelodysplastic syndrome (MDS) and in recent trials in lung and ovarian cancer, lower than maximum tolerated doses of demethylating agents have shown improved efficacy [22–25]. However, our data suggests that TGCTs may be uniquely sensitive to even lower doses of DNMTIs. To examine the *in vivo* effects of guadecitabine on EC cells we established cisplatin resistant tumors in mice. Mice were treated by subcutaneous injections with either 2 mg/kg guadecitabine or vehicle control for 5 days per week for 2 weeks once palpable tumors were observed. This is a “low” dose of guadecitabine that only modestly effects ovarian and liver cancer xenograft progression using a similar treatment schedule [26, 27]. This dose of guadecitabine completely abrogated tumor growth and caused complete tumor regression that was evident up to three weeks after treatment cessation (Figure 4A). This treatment resulted in no whole animal toxicity as assessed by body weight (Figure 4C). While no tumor was evident in most mice treated with guadecitabine at this dose, occasionally it was noted that a small avascular mass was present at the cell injection site that did not progress even when treatment was stopped for 4 weeks (Figure 4B). Preliminary histological analysis indicated that these cells represented differentiated yolk sac and teratoma (not shown). In contrast, control tumors were large, highly vascular and comprised mainly of undifferentiated EC cells that often invaded skeletal muscle (Figure 4B and data not shown).

In order to ascertain whether guadecitabine is effective in suppressing EC tumor cell growth at even lower doses, the effect of 2.5 mg/kg guadecitabine and two, five-fold dose de-escalations of guadecitabine were compared. Guadecitabine was highly effective at inducing complete regression and inhibition of EC tumor growth even at doses of 0.5 and 0.1 mg/kg and was effective in regressing large tumors (Figure 4D). However, it was noted that compared to the 2.0 and 2.5 mg/kg doses, some tumors at the 0.5 and 0.1 mg/kg doses began to grow back beginning at 2 weeks post therapy (Figure 4D).

Since pretreatment of cisplatin resistant EC cells with low concentrations of quadecitabine resensitized the cells to cisplatin treatment we assessed whether this also occurs *in vivo*. The growth of the cisplatin resistant tumors after only one cycle (5 days) of 0.5 mg/kg guadecitabine alone or followed by a single 9.0 mg/kg dose of cisplatin was compared. While treatment with guadecitabine at 0.5 mg/kg alone resulted in incomplete tumor inhibition and regrowth, addition of cisplatin 2 days later resulted in complete and lasting regression of the tumors with no evident toxicity (Figure 4E). This is in contrast to what occurs with two consecutive 9.0 mg/kg injections of cisplatin alone, which resulted in either tumor progression, regrowth or lethal toxicity (Figure 4E). Thus the combination of very low dose guadecitabine and cisplatin is more effective than either treatment alone.

### *In vivo* tumor response is associated with genome wide induction of p53 target and immune-related gene signatures

**Figure 5:**
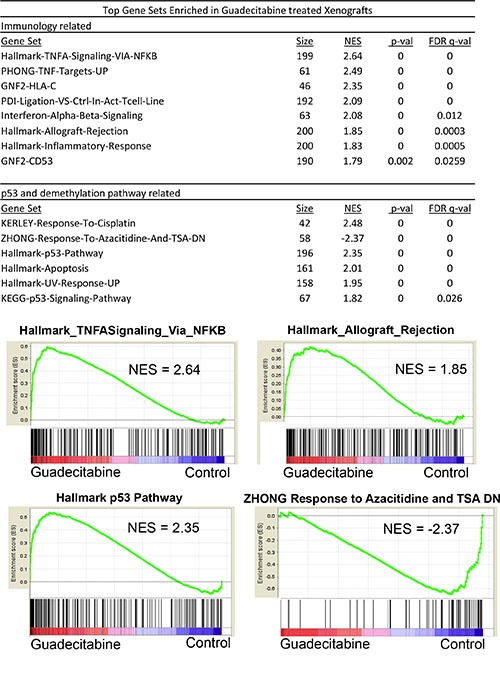
Guadecitabine induces p53 and immune pathway gene expression signatures in EC tumors *in vivo* GSEA analysis of tumors treated *in vivo* for 4 days with 1.5 mg/kg guadecitabine (SGI-110) in biological triplicate revealed that the top gene sets enriched in response to guadecitabine are related to immune or p53 pathway activation. NES is Normalized Enrichment Score.

**Figure 6:**
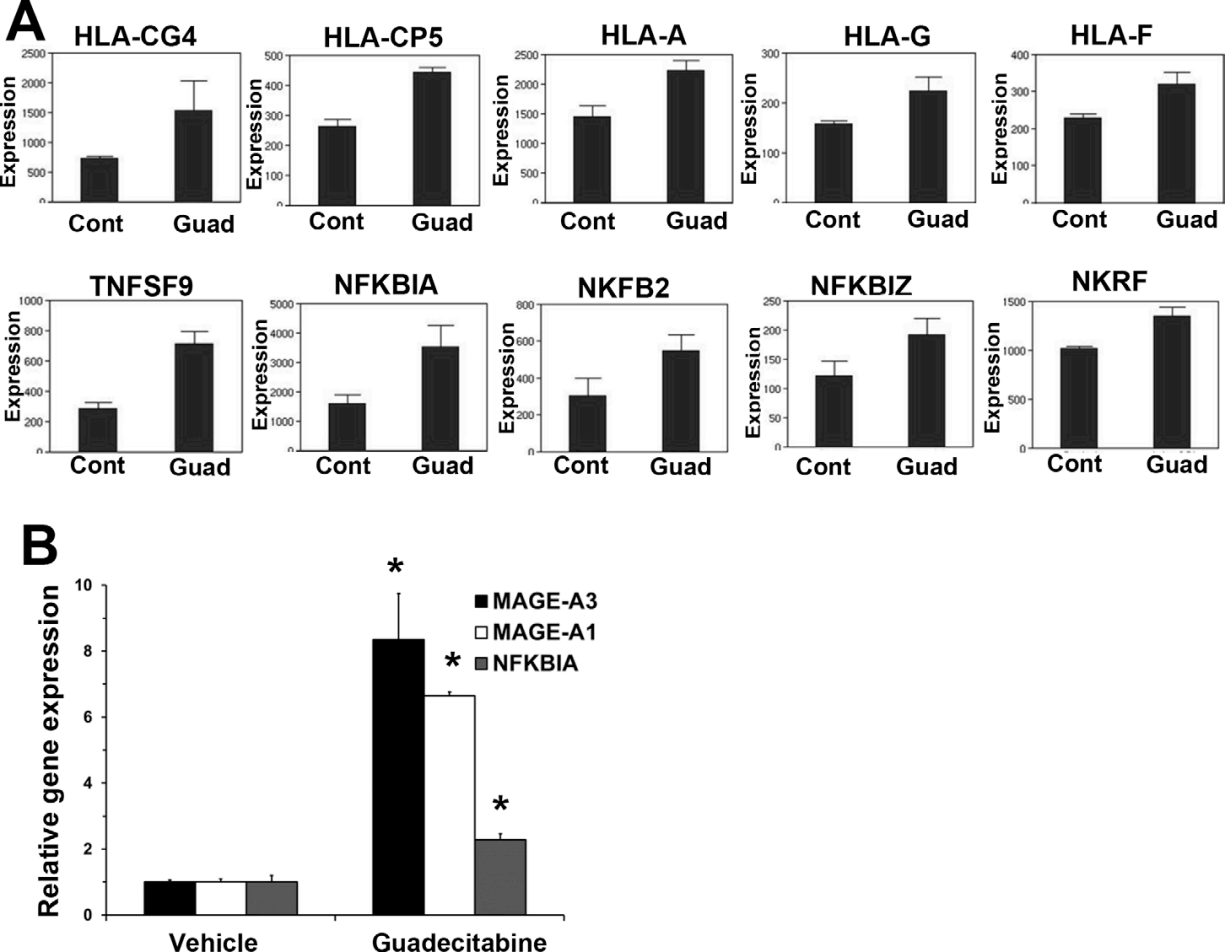
Guadecitabine induces HLA, NFKB and cancer testis antigens in EC tumors (**A**) Microarray results for HLA class I and NFKB pathway genes. (**B**) Real-time PCR analysis of cancer testis antigens IMAGE-A3, IMAGE-A1 and NFKB gene, NFKBIA.

We conducted microarray-based gene expression analysis to compare gene expression changes between control tumors and tumors after only 4 days of 1.5 mg/kg guadecitabine treatment. At this time point guadecitabine treated tumors have not yet begun to regress in size compared to control tumors (Figure 4A). Gene set enrichment analysis (GSEA) indicated that guadecitabine treated tumors induced expression of p53 target genes and related pathways and pathways associated with DNA methylation (Figure 5). However, guadecitabine treated xenografts also were strongly enriched for immune-related gene signatures (Figure 5). This includes signatures associated with TNF, allograft rejection, HLA class C and interferon signaling. Examination of the leading edge genes indicated that these enriched signatures were driven largely by induction of HLA class I and NFKB pathway genes (Figure 6A). DNA demethylating agents have recently been associated with enhancing anti-tumor immunity (28–30). Our data suggests an alternative mechanism of guadecitabine action that may be clinically relevant for the treatment of refractory TGCTs. In this regard, we demonstrated that guadecitabine induced the expression of cancer testis antigens MAGE-A3 and MAGE-A1 in cisplatin resistant EC cells (Figure 6B).

### Low concentration guadecitabine response in cisplatin sensitive and resistant EC is dependent on p53

**Figure 7:**
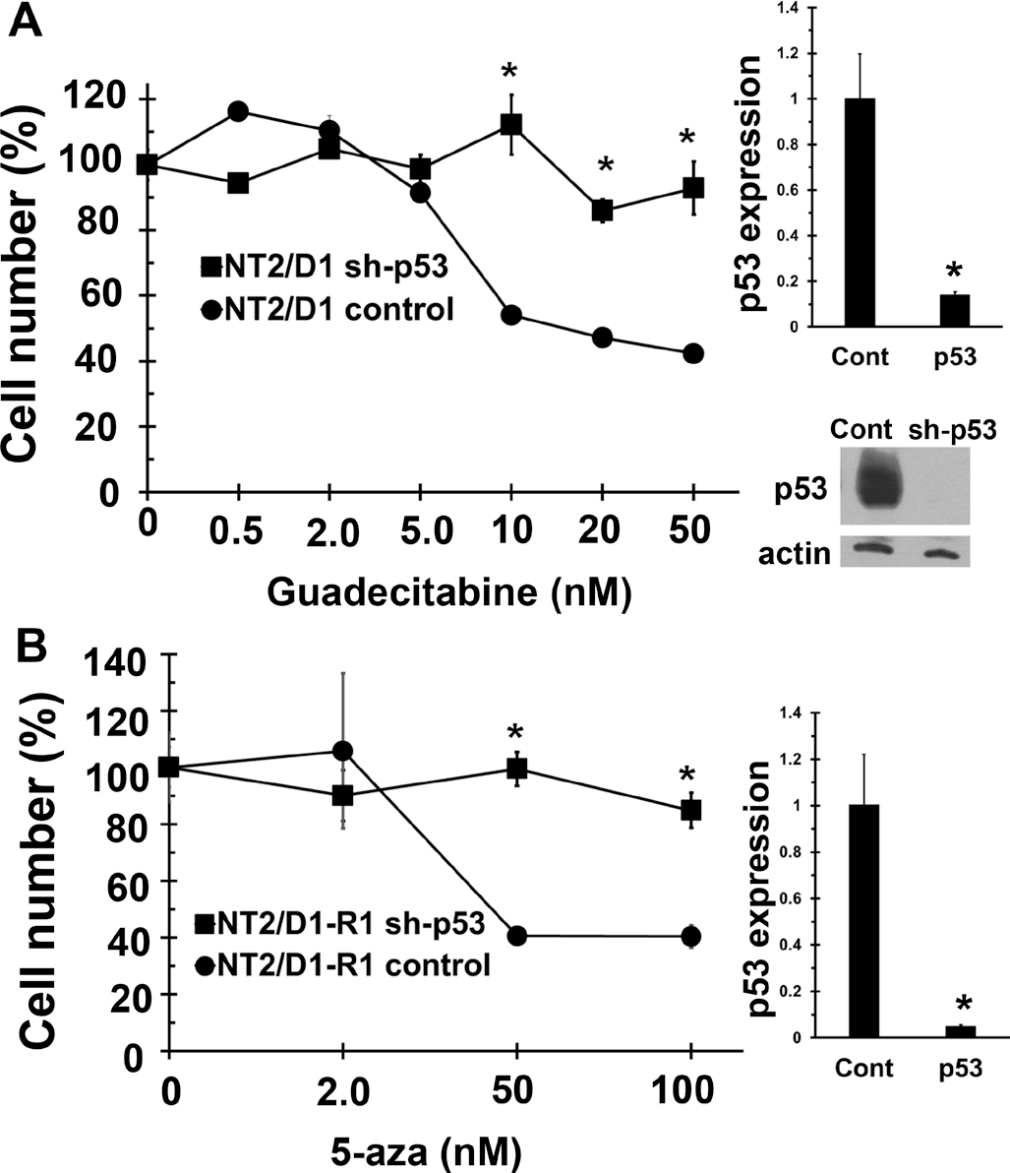
Guadecitabine and 5-aza sensitivity in EC cells is dependent on p53 Knockdown of p53 results in resistance to (**A**) Guadecitabine in NT2/D1 cells and (**B**) 5-aza in NT2/D1-R1 cells. Guadecitabine or 5-aza was added for 3 days to exponentially growing cultures. Viable cell growth and survival were measured. All data points are the average of biological triplicates. Error bars are standard deviation. *p < 0.01 comparing control cell lines with their corresponding shp53 cells. Right, The shp53 knockdown was confirmed by real-time PCR assays. Error bars are standard deviation. *p < 0.01 comparing control cell lines with their corresponding shp53 cells. Western blot showing robust knockdown of p53 in sh-p53 NT2/D1 cells is also included. A similar level of knockdown was also seen in NT2/D1-R1 cells (not shown). Experiments were repeated with similar results.

Since both 5-aza and guadecitabine induce the expression of p53 target genes, the importance of p53 in guadecitabine mediated repression of cell proliferation and survival was assessed. Knockdown of p53 in NT2/D1 and NT2/D1-R1 cells resulted in relative guadecitabine resistance which demonstrates that activation of p53 is important for guadecitabine sensitivity of testicular cancer cells (Figure 7A and 7B).

## DISCUSSION

Acquired resistance to chemotherapy and targeted therapy is currently the single most important impediment to curative treatments of advanced cancers. Due in part to advances in our understanding of epigenetic deregulations in cancer, there has been a recent revisiting of the concept of demethylation therapy, especially the use of demethylation inhibitors to resensitize refractory cancers to no-longer effective therapies [26, 27, 31]. Here we demonstrate that refractory testicular cancer may be particularly sensitive to demethylation therapy. Testicular cancer cells, even those resistant to cisplatin, were highly sensitive to low concentrations of the novel demethylating agent, guadecitabine. Cisplatin resistant TGCT-derived EC were highly sensitive to guadecitabine *in vitro* and *in vivo* and guadecitabine was also able to sensitize cisplatin resistant tumors to cisplatin. Strikingly, as a single agent extremely low doses of guadecitabine completely abolished progression and induced complete regression of cisplatin resistant EC tumors. Mechanistically we demonstrate that these potent antitumor effects of guadecitabine are dependent on DNMT3B and also p53. In *de novo*, genome-wide analysis, we provide evidence that guadecitabine induces early and extensive p53 pathway activation *in vivo* and interestingly also induces immune tumor cell recognition components including HLA class I and cancer testis antigens. Based on the preclinical studies reported here, we suggest that guadecitabine and other demethylation inhibitors may provide a path to overcome acquired drug resistance in testicular cancer, laying a foundation and strong rationale for testing this class of epigenetic drugs in the clinical setting.

Demethylation therapy with 5-aza is approved for the treatment of myelodysplasitic syndrome (MDS) and 5-aza is currently under clinical evaluation for specific leukemia and solid tumors [19, 22–25]. Recent experience with MDS suggests that demethylating agents should be given below the maximum tolerated dose and over many cycles [22]. The scientific basis for lower dose therapy relates to the mechanism of demethylation mediated by the nucleoside analog class of DNMTIs which require incorporation into newly synthesized DNA in contrast to the non-specific toxicity and repression of cell division that occurs at higher doses [17]. This may explain past unsuccessful experiences with demethylation inhibitors given at maximum tolerated doses for solid tumors [31]. For example, a clinical trial using 5-aza-cytidine to treat refractory testicular cancer was unsuccessful in improving outcomes [32]. However, in this trial 5-aza-cytidine was used at high doses as a non-specific cytotoxic agent and not at the low doses that are optimal to inhibit DNA methylation. The trial also did not test whether DNMTI therapy could sensitize refractory TGCTs to cisplatin [32]. Recent studies have also suggested that lower “biologically effective” doses of 5-aza may be an effective clinical strategy to target tumor-initiating cells in solid tumors [23, 24, 33].

Our data demonstrates that testicular cancer cells are uniquely sensitive to extremely low doses of DNMTIs suggesting that these agents can potentially be used at doses with very little toxicity. At first glance it may seem paradoxical that EC cells are highly sensitive to DNA methylation inhibitors but resistant to these inhibitors when one of its targets, DNMT3B is depleted. This may be related to incorporation of 5-aza into newly synthesized DNA when given at low doses resulting in DNMT3B-DNA adduct formation. More studies are required to better define the nature of the DNMT3B-dependent sensitivity of EC cells to demethylating agents.

The mechanism to account for the sensitivity of EC cells to DNMTs is unclear but is likely related to the germ cell origins of TGCTs [3, 34]. Seminomas have very little DNA methylation compared to normal cells and somatic tumors while nonseminomas and EC have an intermediate level and there is some evidence to suggest an association between cisplatin resistance in TGCTs and increased methylation of candidate tumor suppressor genes [14, 15, 35–37]. In addition, teratomas are heavily methylated and resistant to cisplatin. We show here that multiple downstream mechanisms appear to be engaged in the antitumor effect of low concentration guadecitabine in testicular tumors including activation of p53 and repression of pluripotency gene expression.

DNMTIs such as 5-aza are subject to rapid degradation by hydrolytic cleavage and deamination by cytidine deaminase and are unstable after intravenous infusion, limiting their potential as cancer therapeutics [20]. Guadecitabine is a dinucleotide comprised of guanosine and decitabine linked by a phosphodiester bond. Guadecitabine is largely resistant to cytidine deaminase degradation resulting in prolonged *in vivo* drug exposure following small volume subcutaneous administration [19]. This DNMTI was shown to achieve hypomethylation and was well tolerated in primates [38]. In a phase I/II trial in acute myeloid leukemia and MDS patients, guadecitabine was shown to be better tolerated and demonstrated activity in patients who had progressed on 5-aza [19]. While 5-aza is unstable and given intravenously, guadecitabine given subcutaneously has a longer effective half-life and a more extended exposure window compared to intravenous infusion of 5-aza [19].

Aberrant DNA hypermethylation down-regulates the expression of components of the “tumor recognition complex” including HLA class I, tumor-associated antigen, cancer testis antigens and accessory/co-stimulatory molecules in neoplastic cells including cutaneous melanoma and sarcoma cancer cells and DNMTIs have been shown to induce the expression of some of these components. [28–30]. In the current study we provide evidence that guadecitabine induces immune signatures including induction of HLA class I, cancer testis antigens and the NFKB pathway in EC tumors. The contribution of this activation to the dramatic antitumor effects of guadecitabine in immune-compromised mice is likely minor but suggests that enhanced immune recognition of human TGCTs may be an additional beneficial feature of guadecitabine therapy in the clinic.

While the testis is thought to be an immune privileged site, the major clinical problem for testicular cancer is metastatic disease. Whether immune privilege in the testis is mediated primarily by structural features within the testis itself or due to immune tolerance that may persist and extend to testicular cancer cells outside the testis is not known. More studies are needed to determine whether immune privilege would be an impediment to immune-based therapies for metastatic testicular cancer and whether induction of HLA class I and cancer testis antigens by quadecitabine could break this immune tolerance if it exists.

In summary, the *in vitro* and *in vivo* preclinical findings in this report demonstrating the striking and potent activity of guadecitabine in TGCT cells strongly supports further investigation of DNMTI-based therapies for the treatment of testicular cancer. The progression of this basic science discovery to a clinical setting using guadecitabine to sensitize cisplatin refractory TGCTs is currently underway in the form of an open label proof of concept phase I study we are currently conducting (NCT02429466).

## MATERIALS AND METHODS

### Cell culture and drug treatments

All cell lines were cultured in DMEM media with 10% fetal bovine serum supplement plus glutamine and antibiotics. Cells were treated with the indicated concentrations of 5-aza-deoxycytidine (5-aza) or guadecitabine (SGI-110) for 3 days with drug replenished each day. Cisplatin treatments were performed at the concentrations and time points indicated. To assess cell proliferation and survival, Cell-Titre Glo (Promega) assays were performed. Guadecitabine was provided by Astex Pharmaceuticals. All other drugs and chemicals were purchased from Sigma. HCT116 (colon cancer), U20S (osteosarcoma) MCF7 (breast cancer) and NT2/D1 (embryonal carcinoma) cells were obtained from the American Type Culture Collection (ATCC) and authenticated by the ATCC with karyotyping and/or short tandem repeat (STR) profiling. NT2/D1-R1 cells are 10-fold resistant to cisplatin and their characterization and derivation have been previously described [39]. Cells were frozen within 1 month of purchase and used within 2 months after resuscitation. NT2/D1-R1 cells were frozen within 2 months of derivation and used within 2 months after resuscitation.

### Lentiviral shRNA production

NT2/D1-sh84 and NT2/D1-R1-sh84 cells with stable lentiviral silencing of DNMT3B were previously described and have a knockdown of DNMT3B of greater than 90% at the protein level [18]. Lentiviral silencing shRNA targeting p53 (TRCN0000010814) was purchased from Thermo Fisher Scientific along with TRC lentiviral non-targeting shRNA control (RHS6848). The sequence of shp53 is GAGGGATGTTTGGGAGATGTA. Lentiviral stocks were generated from 293T cells as previously described [18]. Cells were cultured with lentiviral stocks for 24 hours and stable pools were selected with 1.0 μg/mL puromycin.

### Real-time PCR analysis

Generation of cDNA was performed with the High Capacity cDNA Reverse Transcription Kit (Applied Biosystems). Real-time polymerase chain reaction (PCR) assays were performed with iTaq Universal SYBR Green Supermix (Bio-Rad Laboratories) and the ddCt method was employed with normalization to GAPDH [40]. Primer sequences are available upon request.

### Mouse tumor assays

All animal studies were approved by the Dartmouth IACUC under protocol spin.mj.1. For mouse studies, 5–8 week old male athymic nude mice (Charles River) were injected subcutaneously in the flank with 5 × 10^6^ cisplatin resistant NT2/D1-R1 cells after resuspension in a 50:50 ratio of DMEM/Matrigel (Corning). Once palpable tumors were detected, tumor volume was measured every two days with calipers using the formula V= (L × W × W)/2. Guadecitabine was diluted in PBS and delivered by subcutaneous injection. Cisplatin was delivered by intraperitoneal injection.

### Gene expression microarray analysis

Tumors were dissected free from stroma and mouse tissue, washed in PBS and immediately placed in RNAlater (Thermo Fisher Scientific). RNA was extracted using an RNeasy Mini Kit (Qiagen) and quality control was performed with the Agilent Bioanalyzer and RNA was quantified with the Qubit Fluorometer (Thermo Fisher Scientific) and stored at -80°C. Expression analysis was performed in triplicate with the Illumina HumanHT-12 v4 bead chip arrays (Illumina) and scanned on the BeadArray Reader (Illumia) according to the manufacturer's instructions. Raw data were normalized (quantile) and analyzed with Genome Studio software (Illumina). GSEA software was downloaded from the Broad website (http://www.broadinstitute.org/gsea/index.jsp). The number of permutations was 1,000 and the permutation type was gene_set. Gene expression microarray data has been submitted to the NCBI GEO repository as GSE90681.

### Statistics

When a value for statistical significance is provided, a two-sample, one-tailed *t*-test assuming unequal variance was performed.
